# Alcohol Intake and Blood Pressure Levels: A Dose-Response Meta-Analysis of Nonexperimental Cohort Studies

**DOI:** 10.1161/HYPERTENSIONAHA.123.21224

**Published:** 2023-07-31

**Authors:** Silvia Di Federico, Tommaso Filippini, Paul K. Whelton, Marta Cecchini, Inga Iamandii, Giuseppe Boriani, Marco Vinceti

**Affiliations:** CREAGEN - Environmental, Genetic and Nutritional Epidemiology Research Center, Section of Public Health, Department of Biomedical, Metabolic and Neural Sciences (S.D.F., T.F., M.C., I.I., M.V.), University of Modena and Reggio Emilia, Italy.; Unit of Cardiology, Department of Biomedical, Metabolic and Neural Sciences (G.B.), University of Modena and Reggio Emilia, Italy.; School of Public Health, University of California Berkeley, CA (T.F.).; Department of Epidemiology, Tulane University School of Public Health and Tropical Medicine, New Orleans, LA (P.K.W.).; Department of Epidemiology, Boston University School of Public Health, MA (M.V.).

**Keywords:** alcohol drinking, blood pressure, cardiovascular risk, meta-analysis, prevention, systematic review

## Abstract

**BACKGROUND:**

Alcohol consumption may increase blood pressure but the details of the relationship are incomplete, particularly for the association at low levels of alcohol consumption, and no meta-analyses are available for nonexperimental cohort studies.

**METHODS:**

We performed a systematic search of longitudinal studies in healthy adults that reported on the association between alcohol intake and blood pressure. Our end points were the mean differences over time of systolic (SBP) and diastolic blood pressure (DBP), plotted according to baseline alcohol intake, by using a dose-response 1-stage meta-analytic methodology.

**RESULTS:**

Seven studies, with 19 548 participants and a median follow-up of 5.3 years (range, 4–12 years), were included in the analysis. We observed a substantially linear positive association between baseline alcohol intake and changes over time in SBP and DBP, with no suggestion of an exposure-effect threshold. Overall, average SBP was 1.25 and 4.90 mm Hg higher for 12 or 48 grams of daily alcohol consumption, compared with no consumption. The corresponding differences for DBP were 1.14 and 3.10 mm Hg. Subgroup analyses by sex showed an almost linear association between baseline alcohol intake and SBP changes in both men and women, and for DBP in men while in women we identified an inverted *U*-shaped association. Alcohol consumption was positively associated with blood pressure changes in both Asians and North Americans, apart from DBP in the latter group.

**CONCLUSIONS:**

Our results suggest the association between alcohol consumption and SBP is direct and linear with no evidence of a threshold for the association, while for DBP the association is modified by sex and geographic location.

High blood pressure (BP) is the most important preventable risk factor for cardiovascular disease.^1–4^ Usual BP levels are influenced by genetic, general environmental, and especially lifestyle factors such as diet composition, body weight, physical activity, and alcohol consumption.^3^ A large body of information links alcohol consumption with level of BP,^5,6^ although there is uncertainty regarding the relationship between alcohol consumption and BP at or below an intake of 2 standard alcoholic drinks per day. Whether this results from a lack of statistical power in studies of low levels of alcohol intake or reflects biological differences at lower compared with higher intakes of alcohol is unclear.^5^

Intervention studies have generally been conducted as short-term randomized controlled trials that investigated the BP effects of reduced alcohol intake in adults with a high alcohol intake.^5,7–9^ The body of evidence detailing the effects of changes in alcohol intake on systolic blood pressure (SBP) and diastolic blood pressure (DBP) in persons who were initially consuming light to moderate amounts of alcohol is limited, with neither systematic reviews or meta-analyses being available on this topic. Longitudinal studies on the association between low intakes of alcohol and BP have been published recently,^10,11^ and a new statistical meta-analytic technique that allows for a comprehensive exploration of dose-response relationships, the so-called 1-stage dose-response meta-analysis for aggregated data, has recently been reported.^12^ This technique allows inclusion of studies in the dose-response analysis even when as few as 2 categories of exposure have been assessed,^13,14^ preserving any study-specific structure of contrasts in the estimation of the dose-response relationship within a single model.^15^ Because of these considerations and the public health importance of the topic, we conducted an analysis of the association between alcohol consumption and BP, including the association at lower levels of alcohol consumption, in nonexperimental cohort studies.

## METHODS

### Data Availability

We conducted this systematic review according to the Preferred Reporting Items for Systematic Reviews and Meta-Analyses guidelines,^16^ after registering it in the PROSPERO international database (no. CRD42022314389). The authors declare that all supporting data are available within the article and its Supplemental Material.

### Search Strategy

We performed a systematic search of the literature in the PubMed and Embase databases to identify longitudinal human studies, published in English or Italian before May 9, 2023, which reported on the association between usual alcohol intake and BP levels. We used the key words alcohol, blood pressure, hypertension to identify the potentially relevant studies. In this manuscript, we focus on the BP end point. Additional details of our search strategy are reported in Table S1.

### Study Selection

A study was considered to be eligible for this meta-analysis if it (1) was based on a cohort or case-cohort investigation; (2) evaluated the relationship between alcohol consumption and change in BP during follow-up; (3) included substantially healthy, adult participants, 4) reported mean BP and the corresponding 95% CIs by baseline alcohol intake categories, or provided the data needed to calculate the corresponding variances. We excluded studies based solely on use of a cross-sectional design, and studies that enrolled participants with previously diagnosed cardiovascular disease, diabetes or cirrhosis. We also excluded studies that only recruited alcoholics, or exclusively investigated binge drinking and effects of acute alcohol consumption.

An assessment of the title, abstract and full-text of potentially eligible studies was conducted independently by 3 authors (S.D.F., M.C., I.I.), with resolution of disagreement by consensus or in consultation with 2 additional authors (T.F. and M.V.).

### Risk of Bias Assessment

Two authors (S.D.F. and T.F.) investigated the risk of bias of eligible studies independently using the Risk of Bias in Nonrandomized Studies of Exposure assessment tool.^17^ Disagreements were resolved by consultation with a third author (M.V.). We considered the following domains for bias assessment: (1) confounding, (2) participant selection, (3) exposure classification, (4) departure from the intended exposure, (5) missing data, (6) outcome ascertainment, and (7) selective reporting. We defined 3 tiers of risk of bias (low, moderate, and high) according to the evaluation of these 7 domains. A study was rated as having an overall low risk of bias if all domains were at low risk, at moderate risk if 1 or 2 domains were at moderate risk of bias, and at high risk when at least 1 domain was at high risk or when 3 or more domains were at moderate risk.

### Data Extraction and Analysis

Three authors (S.D.F., M.C., I.I.) extracted the following data for all the selected studies: (1) study characteristics (first author name, study design, publication year, follow-up), (2) cohort characteristics (country, study cohort, sex, age, number of participants, smokers), (3) exposure (intake assessment), (4) outcome assessments, and (5) potential confounders considered in the multivariable analysis.

We plotted the mean difference of BP over time between the highest and lowest categories of baseline alcohol intake in each study using a forest-plot displaying mean difference and corresponding 95% CIs. We performed a dose-response meta-analysis using a 1-stage approach to explore the possibility of a nonlinear association.^18^ In particular, we used a restricted cubic spline model of SBP and DBP differences with 3 knots at fixed percentiles (10th, 50^th^, and 90th) of baseline alcohol intake based on the restricted maximum likelihood random-effects model.^12^ For this purpose, we extracted the mean or median dose for each exposure category of alcohol intake. If the dose of alcohol intake was open-ended in 1 direction, we imputed a dose 20% more or less than the cut point reported.^19^ Studies that provided mean BP differences for a continuous exposure were excluded from the dose-response analysis. For 2 studies^20,21^ that reported alcohol intake as frequency of drinks/day and drinks/week, respectively, we estimated alcohol intake in grams/day by matching 1 drink to 1 alcohol unit, that is, 12 grams (g) of alcohol. For 1 study^11^ in which alcohol consumption was reported in mL/day, we estimated alcohol intake/day by considering 15 mL of alcohol as equivalent to 12 g of alcohol.^22^ We extracted or computed the SBP and DPB changes over time according to baseline alcohol exposure, using the most adjusted multivariable model available based on inclusion of established and potential confounders.

We conducted analyses stratified by sex and geographic region (Asia and North America), and sensitivity analyses that excluded studies not adjusted for body mass index or smoking status. We also evaluated the effect modification of baseline BP and duration of follow-up on mean BP difference at the end of the study related to usual baseline alcohol intake by use of a meta-regression model. We assessed the heterogeneity of the included studies using the I^2^ statistic for forest-plots, and through graphical overlay of study-specific predicted curves in the dose-response meta-analysis, to show the extent of variation across studies. Finally, we evaluated the likelihood of publication bias using funnel plots and by computing the Egger’s test.

## RESULTS

**Figure 1.**
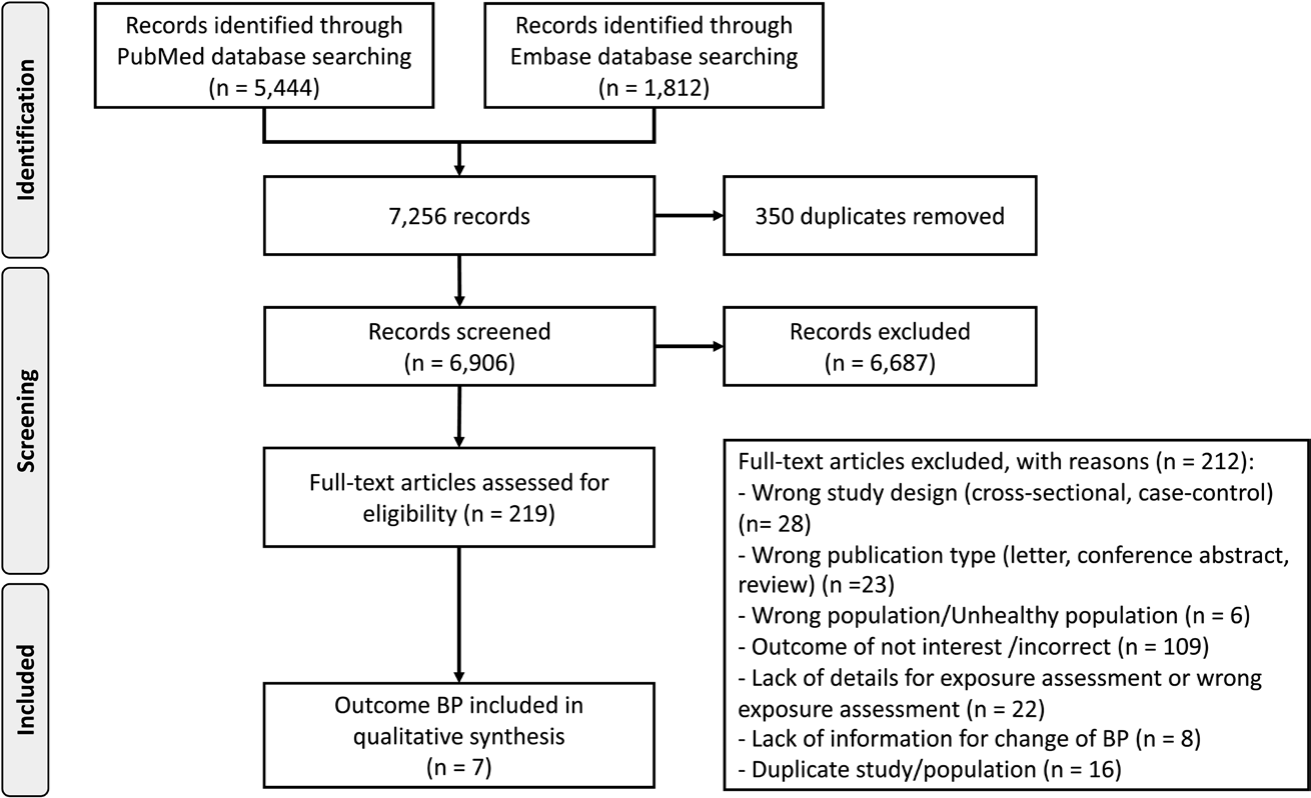
**Preferred reporting items for systematic reviews and meta-analyses flow chart for study selection.** BP indicates blood pressure.

We present our Preferred Reporting Items for Systematic Reviews and Meta-Analyses flow chart in Figure 1. We retrieved 7256 study reports, 350 of which were duplicate publications. We excluded an additional 6906 studies after title and abstract screening. We excluded 212 of the remaining 219 full-text papers. The agreement between reviewers was 95% for title and abstract screening and 65% for full-text evaluation. Reasons for exclusion after full-text evaluation were use of a nonlongitudinal study design (n=28), irrelevant publication type (eg, reviews, commentaries; n=23), selection of an unhealthy population (n=6), failure to report our study outcome (n=109), lack of sufficient exposure details (n=22), lack of information for BP change (n=8), or duplicate publication (n=16). Seven reports met the requirements for inclusion in our dose-response meta-analysis (Table 1). These reports were published between 1997 and 2021 and were conducted in either North America (n=3) or Asia (n=4). Overall, they included 19 548 participants, of whom 12 701 (65%) were men and 6650 were women. Three studies were conducted exclusively in men, 2 included both men and women combined, and 2 stratified the analysis by sex. Four studies included smoking status as a potential confounder in their multivariable analysis model, and 5 included body mass index. The shortest duration of follow-up was 4 years and the longest was 12 years, with a median value of 5.3 years. Details of the risk of bias assessment are reported in Table S2. Two studies were categorized as having a low risk of bias,^11,24^ 4 as having a moderate risk,^20,21,23,26^ and the remaining one as having a high risk of bias.^25^

**Table 1.**
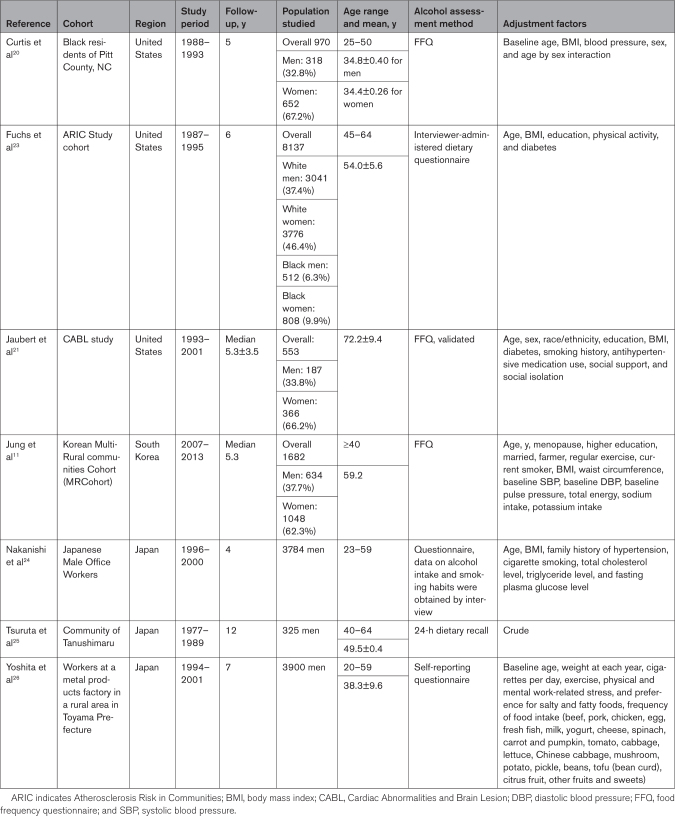
Characteristics of the 7 Studies Included in the Dose-Response Meta-Analysis

As shown in Figure S1, forest-plots comparing the highest versus the lowest category of alcohol intake demonstrated an increased mean difference for SBP and DBP (mean difference, +4.30 mm Hg [95% CI, −2.76 to +5.85] with I^2^=67.11%, and +2.42 mm Hg [95% CI, +1.13 to +3.71] with I^2^=78.45%, respectively).

**Figure 2.**
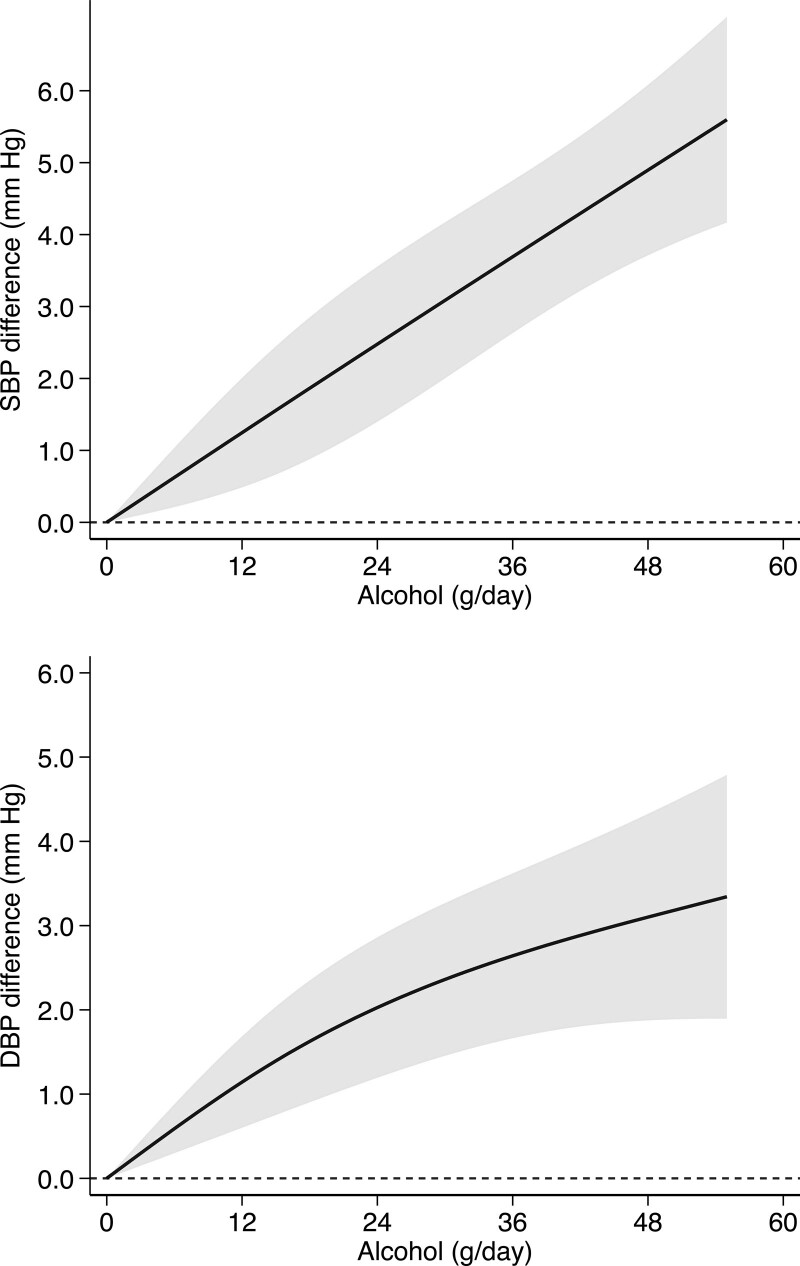
**Dose-response relationship between baseline alcohol intake and systolic blood pressure (SBP) and diastolic blood pressure (DBP) in all cohort studies.**^11,20,21,23–26^ Spline curve (solid line) with 95% confidence limits (gray area).

In Figure 2, we report the results of the dose-response meta-analysis in the entire population. There was an almost linear positive association between baseline alcohol consumption and BP change over time, with the slope being steeper and more linear for SBP than for DBP. Usual SBP and DBP were 1.25 mm Hg (95% CI, +0.49 to +2.01) and 1.14 mm Hg (95% CI, +0.60 to +1.68) higher, respectively, for a 12 g/day greater daily consumption of alcohol compared with no alcohol consumption. The corresponding SBP and DBP differences for a daily alcohol consumption of 24 g/day were 2.48 (95% CI, +1.40 to +3.56) and 2.03 (95% CI, 1.19 to +2.86) mm Hg, and for 48 g/day were 4.90 (95% CI, +3.71 to +6.08) and 3.10 (95% CI, +1.88 to +4.33) mm Hg.

**Figure 3.**
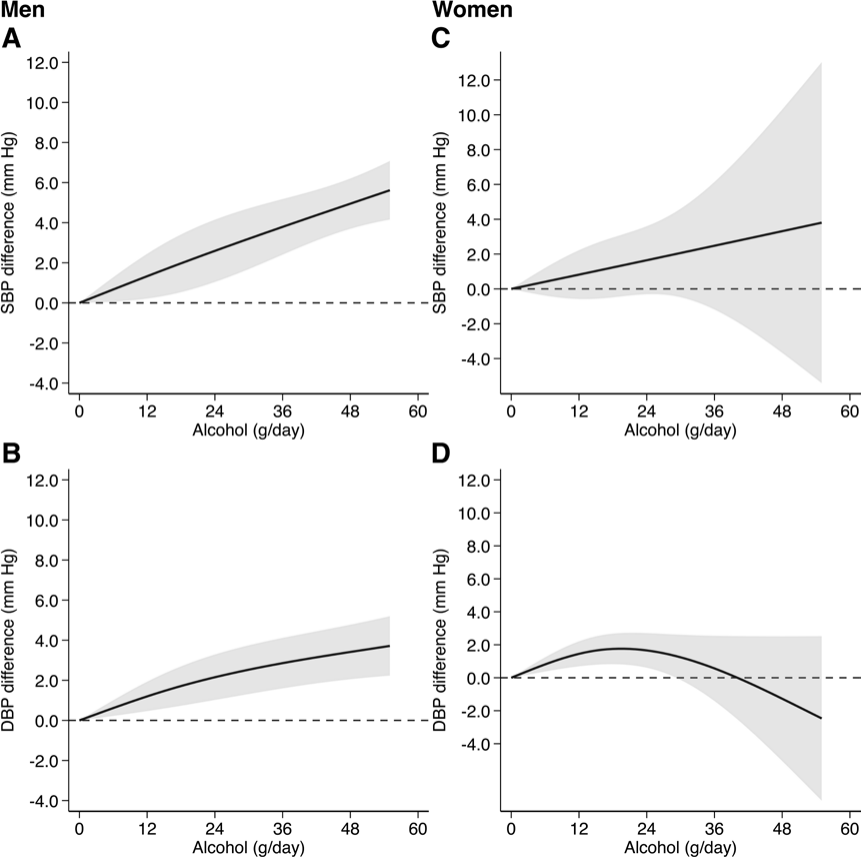
**Dose-response relationship of baseline alcohol intake with systolic blood pressure (SBP) and diastolic blood pressure (DBP) divided by sex.** Studies conducted in men (**A** and **B**)^23–27^ and women (**C** and **D**).^23,27^ Spline curve (solid line) with 95% confidence limits (gray area).

In a sex-specific analysis, based on 5 studies with 12 196 men and 2 studies with 5632 women, the association between alcohol intake and BP was almost linear but steeper in men compared with women, with the exception that in women DBP was not directly associated with alcohol consumption and showed some evidence for an inverted *U*-shape pattern (Figure 3). In men, the SBP difference was 1.33 mm Hg (95% CI, +0.22 to +2.44) for an alcohol intake of 12 g/day compared with no alcohol consumption and 4.95 mm Hg (95% CI, +3.70 to +6.20) for an intake of 48 g/day. In women, the SBP was 0.82 mm Hg (95% CI, −0.58 to +2.22) higher for an alcohol intake of 12 g/day compared with no alcohol consumption and 3.31 mm Hg (95% CI, −3.67 to +10.30) higher for an intake of 48 g/day. The corresponding DBP differences for men were 1.20 mm Hg (95% CI, +0.47 to +1.93) for 12 grams of alcohol and 3.41 mm Hg (95% CI, +2.06 to +4.77) for 48 g/day of alcohol. In women, DBP was 1.45 mm Hg (95% CI, +0.70 to +2.20) higher for an alcohol intake of 12 g/day and was −1.27 mm Hg (95% CI, −5.04 to +2.51) lower for an alcohol intake of 48 g/day.

**Figure 4.**
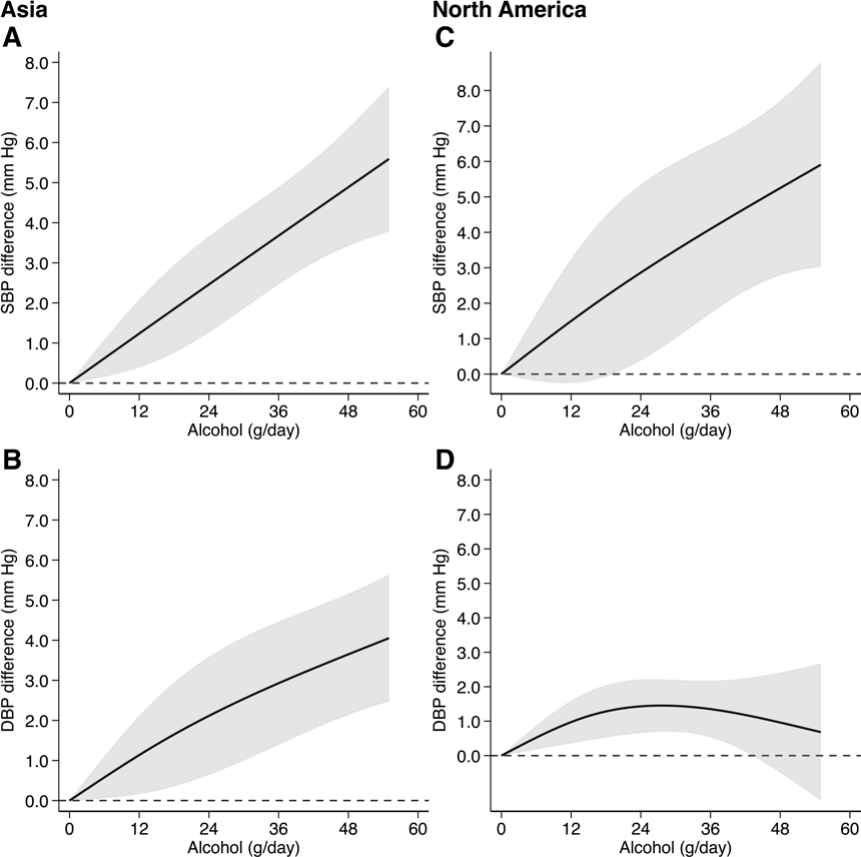
**Dose-response relationship of baseline alcohol intake with systolic blood pressure (SBP) and diastolic blood pressure (DBP) divided by region.** Studies conducred in Asia (**A** and **B**)^11,24–26^ and North America (**C** and **D**).^20,21,23^ Spline curve (solid line) with 95% confidence limits (gray area).

In subgroup analysis by region, we observed similar patterns to those identified for the overall analysis in the studies conducted in Asia (Figure 4). Conversely, after pooling the studies performed in North America, we identified a positive, almost linear association between alcohol intake and SBP, whereas for DBP there was a positive association for an alcohol intake up to 24 g/day but at higher intakes the association flattened and tended to decrease (Figure 4). In the Asian studies, the SBP differences at daily alcohol consumption levels of 12 and 48 g/day were 1.24 mm Hg (95% CI, +0.39 to +2.08) and 4.89 mm Hg (95% CI, +3.42 to +6.35), while the corresponding differences for DBP were 1.14 mm Hg (95% CI, +0.16 to +2.11) and 3.65 mm Hg (95% CI, +2.14 to +5.16). In the North American studies, the SBP changes at daily alcohol consumption levels of 12 and 48 g/day were 1.50 mm Hg (95% CI, −0.25 to +3.24) and 5.25 mm Hg (95% CI, +2.79 to +7.71), while the corresponding changes for DBP were 0.97 mm Hg (95% CI, +0.36 to +1.59) and 0.96 mm Hg (95% CI, −0.49 to +2.41).

Sensitivity analyses after exclusion of studies that did not adjust for smoking or body mass index, respectively, showed associations that were similar to the overall patterns (Figures S2 and S3). Assessment of study-specific curves also showed substantial homogeneity of the results for SBP, while for DBP a higher variability emerged (Figure S4).

There was a positive linear association between baseline level of BP and change in BP during follow-up related to usual alcohol intake at baseline (Figure S5). Similarly, duration of follow-up was positively associated with change in SBP during follow-up related to usual alcohol intake at baseline, with the slope being steeper for change in SBP than for change in DBP (Figure S6). Funnel plots showed little evidence of publication bias, as was also indicated by the Egger test (coefficient for SBP, 0.78 [95% CI, −1.12 to 2.69], and for DBP, 0.09 [95% CI, −2.43 to 2.60]; Figure S7).

## DISCUSSION

The main finding of our meta-analysis was that usual alcohol consumption at baseline was positively associated with change in SBP over time in both men and women, with a substantially linear pattern, although the amount of change associated with a limited amount of alcohol intake (such as one serving/day) was rather small. Our findings suggest that in longitudinal analysis there is no threshold below which no association exists between alcohol consumption and a higher SBP. Consequently, consumption of any alcohol should be considered a risk factor for high SBP, with important preventive and therapeutic implications.

Our findings of a linear positive association between usual baseline alcohol consumption and BP changes during the follow-up compared with baseline values do not appear to hold for DBP in women and in North Americans, with some evidence for an inverted *U*-shaped pattern in both settings. In the stratified analysis restricted to women, there was a threshold of alcohol intake at around 40 g/day above which there was unexpectedly no further increase in DBP and even a slightly lower level of DBP. Likewise, in women the strength of the positive association between alcohol consumption and prospective changes in SBP was less than that observed in men, although the association was still substantially linear. This sex-related difference in the pattern of the association may indicate a causal role of factors such as sex hormones or may have resulted from a bias such as enhanced misclassification of the exposure and outcome values in women consuming high levels of alcohol. Only 2 studies stratified their results by sex,^11,23^ a fact that substantially decreases the statistical stability and precision of the sex-specific results, particularly in women. Moreover, average alcohol intake differed substantially in the 2 available studies for women, with the median value in nonabstainers being 1.52 g/day in one of the 2 studies^11^ whereas the dose ranged from 0 to >30 g/day in the other.^23^ This affected the precision and reliability of our stratified analyses in women at high alcohol exposures and increased the heterogeneity of the results in this subgroup. We have no clear explanation for the unexpected differences in the DBP associations in women who consumed large amounts of alcohol, compared with our consistently positive associations between alcohol intake and SBP in both sexes. A recent cohort study conducted in China, which was not eligible for inclusion in the present meta-analysis because it lacked suitable data, provided no evidence for a smaller change of DBP in light or moderate drinkers, compared with nondrinkers, in a sex-adjusted multivariable model.^10^

While we are not aware of systematic reviews or meta-analyses assessing the relationship between usual alcohol consumption at baseline and subsequent BP changes over time, 2 studies have investigated the association between alcohol intake and risk of hypertension, also focusing on possible sex- and region-specific effects.^27,28^ The first review found a positive correlation between alcohol consumption and risk of hypertension in men, with no evidence of a threshold.^28^ Conversely, the association was *J*-shaped in women, with a slightly decreased risk for intakes of 1 to 2 drinks/day compared with abstainers, and an enhanced risk of hypertension at higher levels of alcohol consumption. The second systematic review which only addressed the relationship in men, reported a positive, dose-dependent association between alcohol intake and hypertension risk which was stronger in Asian men compared with their Western counterparts.^27^ The latter report results tended to mirror our results.

The biological basis for the different pattern of the prospective association between alcohol intake with SBP and DBP in Asians compared with North Americans may be related to known differences in alcohol metabolizing enzymes, such as alcohol dehydrogenase and aldehyde dehydrogenase, between Asians and Westerners.^29^ However, it is unclear why such different genetic backgrounds would only affect DBP and not SBP. A slightly higher risk of hypertension following usual consumption of high levels of alcohol in Asians compared with Westerners has been reported in a previous meta-analysis.^27^

Our meta-regression analysis that investigated the effects modification of baseline BP and on BP changes during follow-up identified a substantially linear positive association suggesting the potential for greater reductions in BP with cessation of alcohol intake in those with a higher starting level of BP. Also, the slightly positive association of duration of follow-up with change in BP during follow-up highlights the potential for bigger reductions in BP with prolonged reduction or abstinence from alcohol.

To the best of our knowledge, this is the first dose-response meta-analysis assessing the relationship between usual baseline alcohol consumption and BP levels in nonexperimental cohort studies, this being its major strength. Our findings are strengthened by exclusive use of data from longitudinal studies conducted in generally healthy adults, thus reducing the risk of reverse causation. Furthermore, we limited our analysis to studies that excluded participants with a history of cardiovascular disease, diabetes, alcoholism, or binge drinking at baseline, to address the relationship between usual baseline alcohol consumption and BP over time with greater validity in healthy individuals. Finally, the overall positive association between alcohol consumption and BP was unchanged even after exclusion of studies that failed to adjust for smoking or body mass index, suggesting that our findings were not due to inadequate adjustment for the main known confounding factors.

Two previous systematic reviews and meta-analyses that explored the effects of experimentally-induced differences in usual alcohol intake on BP have been published,^5,8^ as well as 1 additional investigation of very short-term (hours) administration of alcohol on SBP and DBP compared with placebo.^7^ One of the 2 meta-analyses of clinical trials identified no effect on SBP or DBP when the baseline average participant intake of alcohol was ≤2 drinks per day, whereas a dose-dependent reduction in BP was noted in those with a higher baseline alcohol intake.^5^ The second report, which included a smaller number of trials and relied on a meta-regression linear model, found the percentage of alcohol consumption reduction to be strongly and positively associated with corresponding reductions in both SBP and DBP, with no substantial effect of the duration of the intervention.^8^ That meta-analysis identified no threshold for the effect of a reduction of alcohol consumption on BP, a finding that is generally consistent with our results based on observational studies and use of nonlinear methods of analysis. The trials considered were however generally of short duration (4–8 weeks). In contrast, follow-up in the cohort studies included in our meta-analysis ranged from 4 to 12 years, a major strength compared the meta-analyses based on experimental findings.

In our meta-analysis, a daily intake of alcohol as little as 12 g/day was associated with a corresponding average SBP difference compared with nondrinkers of 1.25 mm Hg (95% CI, +0.49 to +2.01), a change that on a population basis might have a meaningful adverse impact on cardiovascular morbidity.^30–34^

It is well known that small reductions in BP, which can be achieved by changes in lifestyle such as a reduction in alcohol consumption, can prevent hypertension and are likely to prevent cardiovascular events. For example, in the Multiple Risk Factor Intervention Trial (MRFIT) Screenees an average SPB that was lower by 2 mm Hg was associated with lower annual mortality from stroke, coronary heart disease, and all-causes of 6%, 4%, and 3%, respectively.^32^ In the Atherosclerosis Risk in Communities (ARIC) Study, a 1 mm Hg lower level of SBP was associated with 13.5 and 9.0 fewer coronary heart disease events per 100,000 person-years in African Americans and Whites, respectively.^35^ The corresponding estimates for heart failure were 20.3 and 13.3, and for strokes were 12.1 and 4.8. In the Chronic Renal Insufficiency Cohort (CRIC) Study, a difference in urinary sodium excretion similar to what was achieved during long-term follow-up in the Trials of Hypertension Prevention (TOHP), Phase 1 (18 months) and Phase 2 (36 months) was associated with incident cardiovascular disease (hazard ratio, 95% CI, of 1.10, 1.05 – 11.16 for a 1000 mg higher level of urinary sodium), including incident stroke and heart failure events.^36–38^

We acknowledge several limitations of our meta-analysis. First, the number of available studies was relatively small, particularly for the sex-specific analyses in women, who were also characterized by a limited range of alcohol intake. This increased the heterogeneity of the study results, as shown within the forest-plots that compared the highest and lowest alcohol intake categories, and the study-specific dose-response curves. In addition, we were unable to investigate the association between alcohol consumption and BP changes by age, since only 1 study provided stratification by this factor.^24^ However, age was taken into consideration as a potential confounder within the included studies. Other potential limitations relate to potential exposure misclassification due to the lack of validation of the questionnaires used to assess alcohol intake in 2 of the studies, and the use of conversions to derive daily alcohol intake from the available consumption data in 2 studies. In addition, our assessment of usual alcohol intake was derived from consumption at baseline and we cannot exclude the possibility that some participants may have changed their alcohol consumption during the follow-up period. This type of exposure misclassification could have contributed to the heterogeneity of our results. Similarly, none of the included studies reported stratified analysis by the types of alcohol consumed, precluding our investigation of the potential for different effects on BP based on the consumption of wine, beer or other types of alcoholic drinks. Overall, these limitations might explain some of the heterogeneity for our estimates, especially for DBP. In addition, the quality of the studies that met our eligibility requirements was quite variable, with only 2 of them being categorized as having a low risk of bias. Finally, the nonexperimental design of the studies did not allow us to exclude the possibility of residual confounding, although a relevant bias due to key unmeasured confounders did not appear to be likely. Recognizing these limitations, our study had several important strengths, including our ability to study alcohol consumption associations with BP over much longer periods and with greater precision, especially at lower intakes of alcohol, compared to what is possible using the available clinical trials. Also, the range of alcohol consumption that could be studied in our meta-analysis was much greater than in the corresponding clinical trials meta-analyses reports. Finally, we were able to make better informed public health inferences by restricting our analysis to cohort studies and focusing on the study of alcohol consumption in relatively healthy adults.

Overall, our review indicates that the relationship between alcohol intake and BP is almost linear with no evidence of a threshold for the association. Future research should assess the association in women and in different age groups, both of which are currently characterized by limited availability of relevant data. Likewise, it would be helpful to have studies that assess the possible differential roles of types of alcoholic beverages.

## ARTICLE INFORMATION

### Sources of Funding

None.

### Disclosures

G. Boriani reported speaker’s fees from Bayer, Daiichi Sankyo, Janssen, Sanofi, for activities outside the scope of the submitted work.

## Supplementary Material


