# Supplementary material for: Alcohol Intake and Blood Pressure Levels: A Dose-Response Meta-Analysis of Nonexperimental Cohort Studies
Source: Hypertension. 2023 Jul 31;80(10):1961–9. doi: 10.1161/HYPERTENSIONAHA.123.21224 (PMC10510850; doi:10.1161/HYPERTENSIONAHA.123.21224)
Supplement: Supplementary file 1 [file hyp-80-1961-s001.docx]

**Title:** Alcohol intake and blood pressure levels: a dose-response meta-analysis of non-experimental cohort studies

**Authors:** Silvia Di Federico, MD^1^, Tommaso Filippini, MD, PhD^1,2^, Paul K. Whelton, MB, MD, MSc^3^, Marta Cecchini, MD^1^, Inga Iamandii, MD^1^, Giuseppe Boriani, MD, PhD^4^, Marco Vinceti, MD, PhD^1,5^

**Affiliations:**

^1^CREAGEN - Environmental, Genetic and Nutritional Epidemiology Research Center, Section of Public Health, Department of Biomedical, Metabolic and Neural Sciences, University of Modena and Reggio Emilia, Modena, Italy

^2^School of Public Health, University of California Berkeley, Berkeley, CA, USA

^3^Department of Epidemiology, Tulane University School of Public Health and Tropical Medicine, New Orleans, LA, USA

^4^Unit of Cardiology, Department of Biomedical, Metabolic and Neural Sciences, University of Modena and Reggio Emilia, Modena, Italy

^5^Department of Epidemiology, Boston University School of Public Health, Boston, MA, USA

**Supplemental Table S1.** Details of literature search on online databases.

| **Database** | **Search string** |
| --- | --- |
| PubMed | (ethanol[MH] OR alcohol[tiab] OR ethanol[tiab] OR "Alcoholic Beverages"[Mesh] OR "Alcoholic Beverage"[tiab]) AND ("Hypertension"[Mesh] OR Hypertension[tiab] OR 'blood pressure'[tiab] OR 'pressure, blood'[tiab] OR 'vascular pressure'[tiab] OR "Blood Pressure"[MeSH Terms] OR Stroke[MH] OR stroke[tiab]) AND humans[MH] AND ("Cohort Studies"[Mesh] OR cohort[tiab] OR case-cohort[tiab] OR prospective[tiab]) AND (english[Filter] OR italian[Filter]) |
| Embase | ('alcohol'/exp OR 'ethanol' OR 'alcoholic beverage'/exp OR 'alcoholic drink' OR 'liquor (alcohol)') AND ('hypertension'/exp OR 'blood pressure'/exp OR 'cerebrovascular accident'/exp) AND ('cohort analysis'/exp OR 'longitudinal study'/exp OR 'prospective study'/exp) AND [english]/lim AND [humans]/lim AND ([english]/lim OR [italian]/lim) |

**Supplemental Table S2**. Risk of bias (RoB) of included studies.

| **Studies** | **Bias due to confounding** | **Bias in selecting participants in the study** | **Bias in exposure classification** | **Bias in departure from intended exposure** | **Bias due to missing data** | **Bias in outcome measurement** | **Bias in selection of reported results** | **Study-level RoB Judgment** |
| --- | --- | --- | --- | --- | --- | --- | --- | --- |
| Curtis 1997 ^20^ | Moderate | Low | Low | Low | Low | Low | Low | Moderate |
| Fuchs 2001 ^23^ | Moderate | Low | Low | Low | Moderate | Low | Low | Moderate |
| Jaubert 2014 ^21^ | Low | Low | Low | Low | Moderate | Low | Low | Moderate |
| Jung 2021 ^11^ | Low | Low | Low | Low | Low | Low | Low | Low |
| Nakanishi 2002 ^24^ | Low | Low | Low | Low | Low | Low | Low | Low |
| Tsuruta 2000 ^25^ | High | Low | Moderate | Low | High | Low | Low | High |
| Yoshita 2005 ^26^ | Low | Low | Moderate | Low | Moderate | Low | Low | Moderate |

**Supplemental Figure S1.** Forest-plot comparing the highest versus the lowest alcohol intake and systolic blood pressure (SBP) and diastolic blood pressure (DBP). MD: mean difference; CI: confidence interval; M: men; W: women; BM: Black men; BW: Black women; WM: White men; WW: White women.

******

**Supplemental Figure S2.** Dose-response relationship between baseline alcohol intake and systolic blood pressure (SBP) and diastolic blood pressure (DBP) with exclusion of studies not adjusted for smoking ^11, 21, 24, 26^. Spline curve (solid line) with 95% confidence limits (grey area).


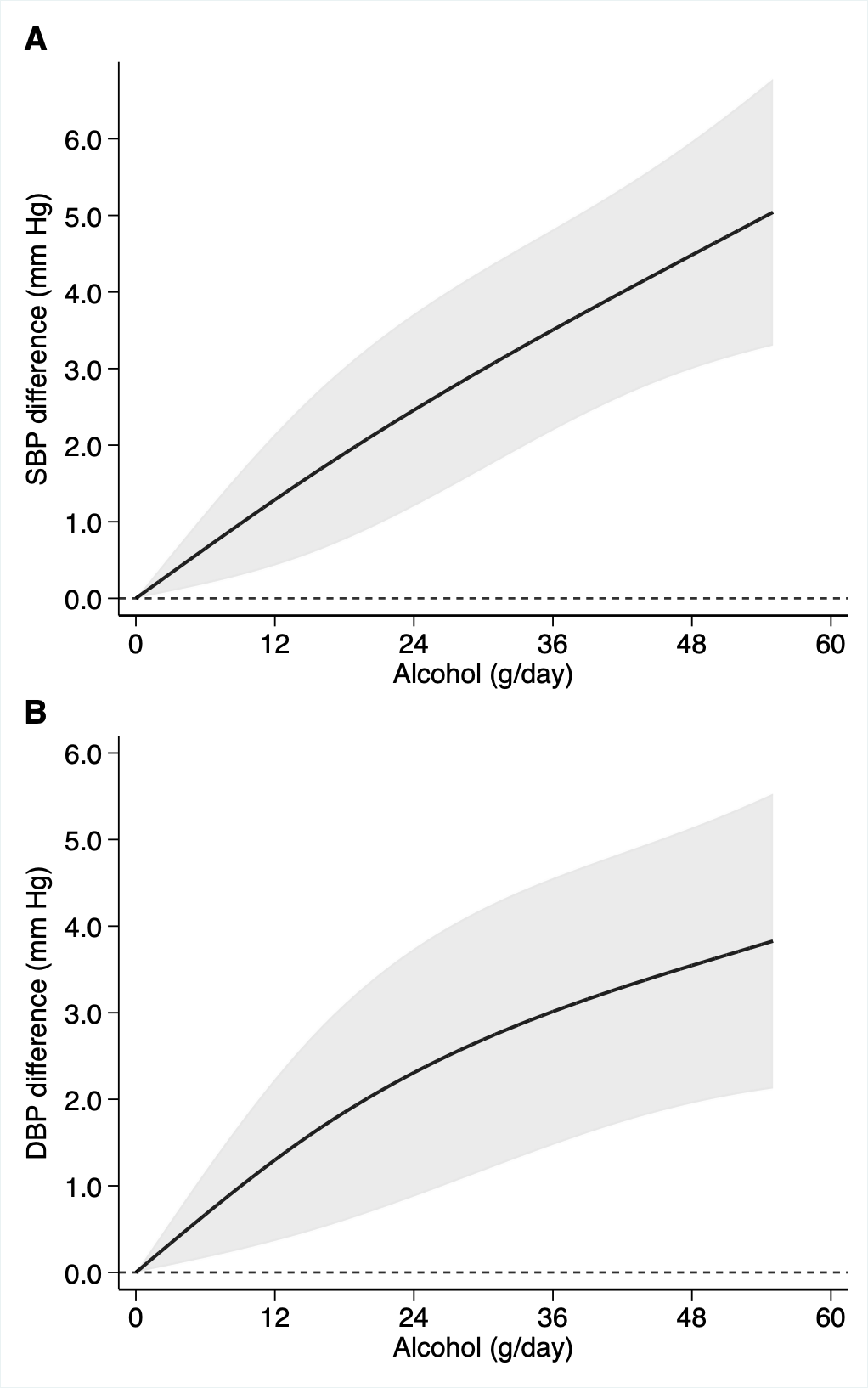


**Supplemental Figure S3.** Dose-response relationship between baseline alcohol intake and SBP (systolic blood pressure) and DBP (diastolic blood pressure) with exclusion of studies not adjusted for body mass index. ^11, 20, 21, 23, 24^. Spline curve (solid line) with 95% confidence limits (grey area).


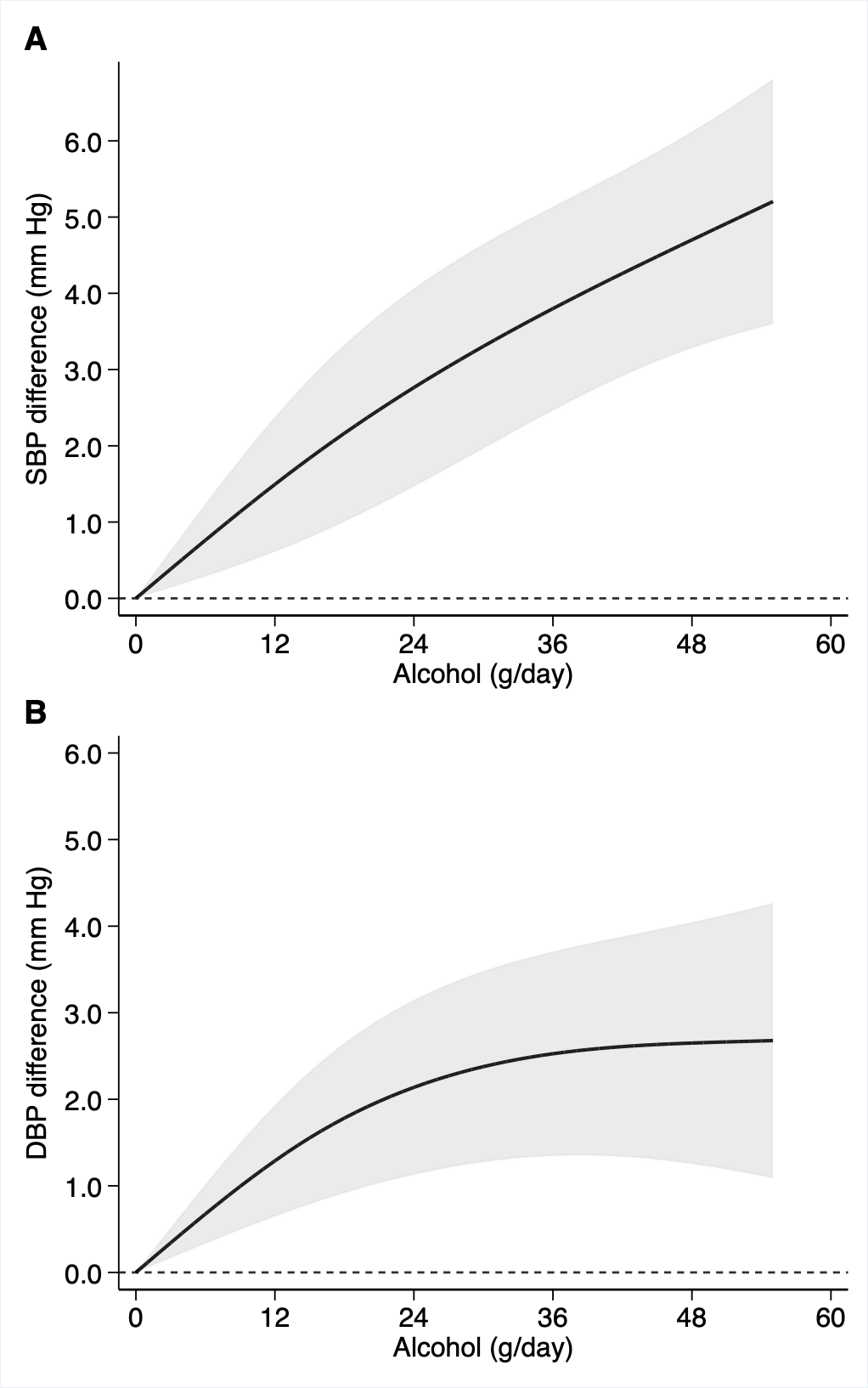


**Supplemental Figure S4.** Sensitivity analysis of the dose-response relationship between baseline alcohol intake and SBP (systolic blood pressure) and DBP (diastolic blood pressure) with study-specific curves ^11, 20, 21, 23-26^. Spline curve (solid line) with 95% confidence limits (grey area). Dark grey curves indicate study-specific trends.


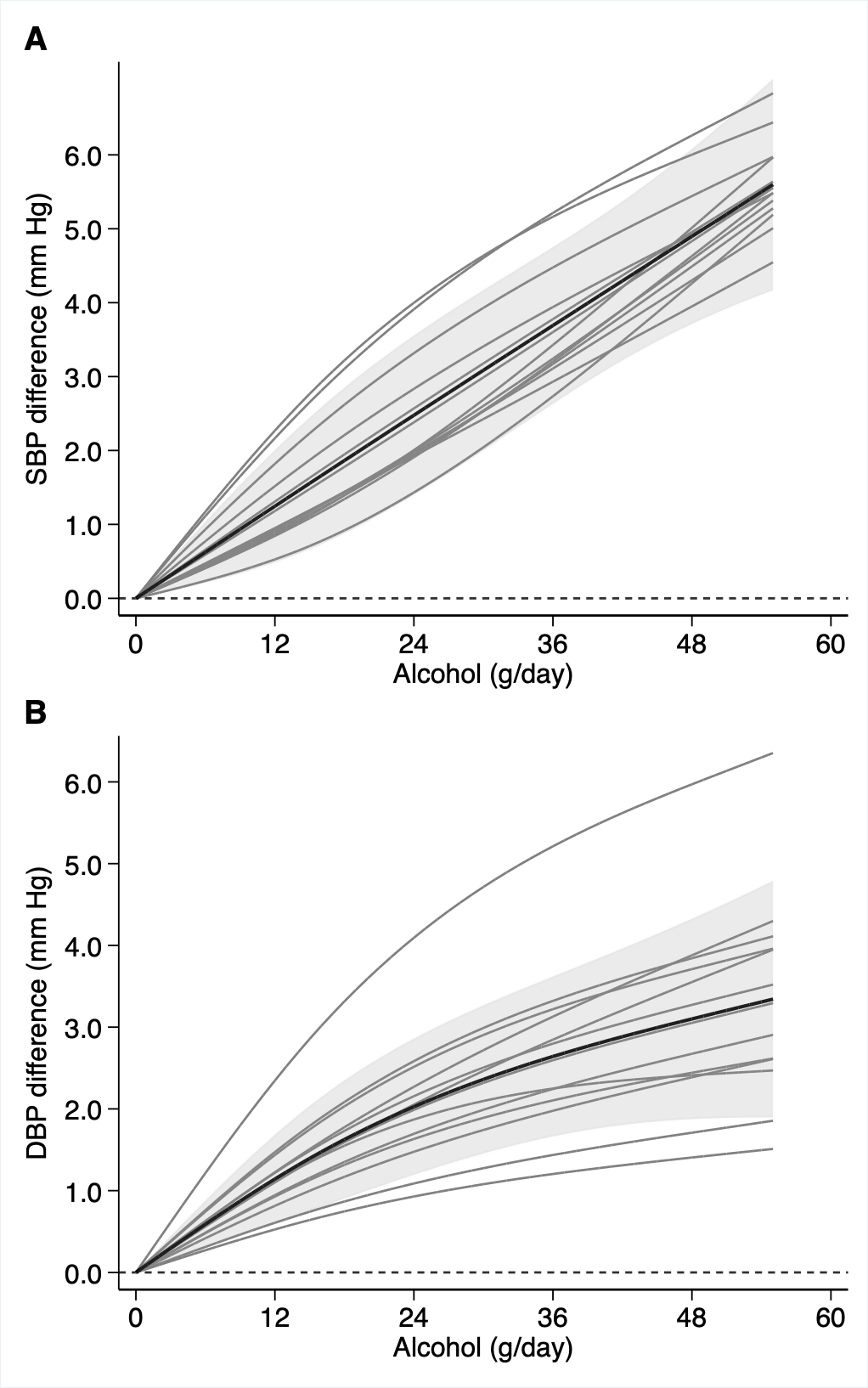


**Supplemental Figure S5**. Bubble plots of meta-regression analysis between baseline SBP (systolic blood pressure) and DBP (diastolic blood pressure) on SBP and DBP changes related to usual alcohol intake at baseline ^11, 20, 23-26^. Hole circles (“bubbles”) are proportional to study weights. Dash lines indicates the predicted regression line with grey areas of confidence-interval bands.

**
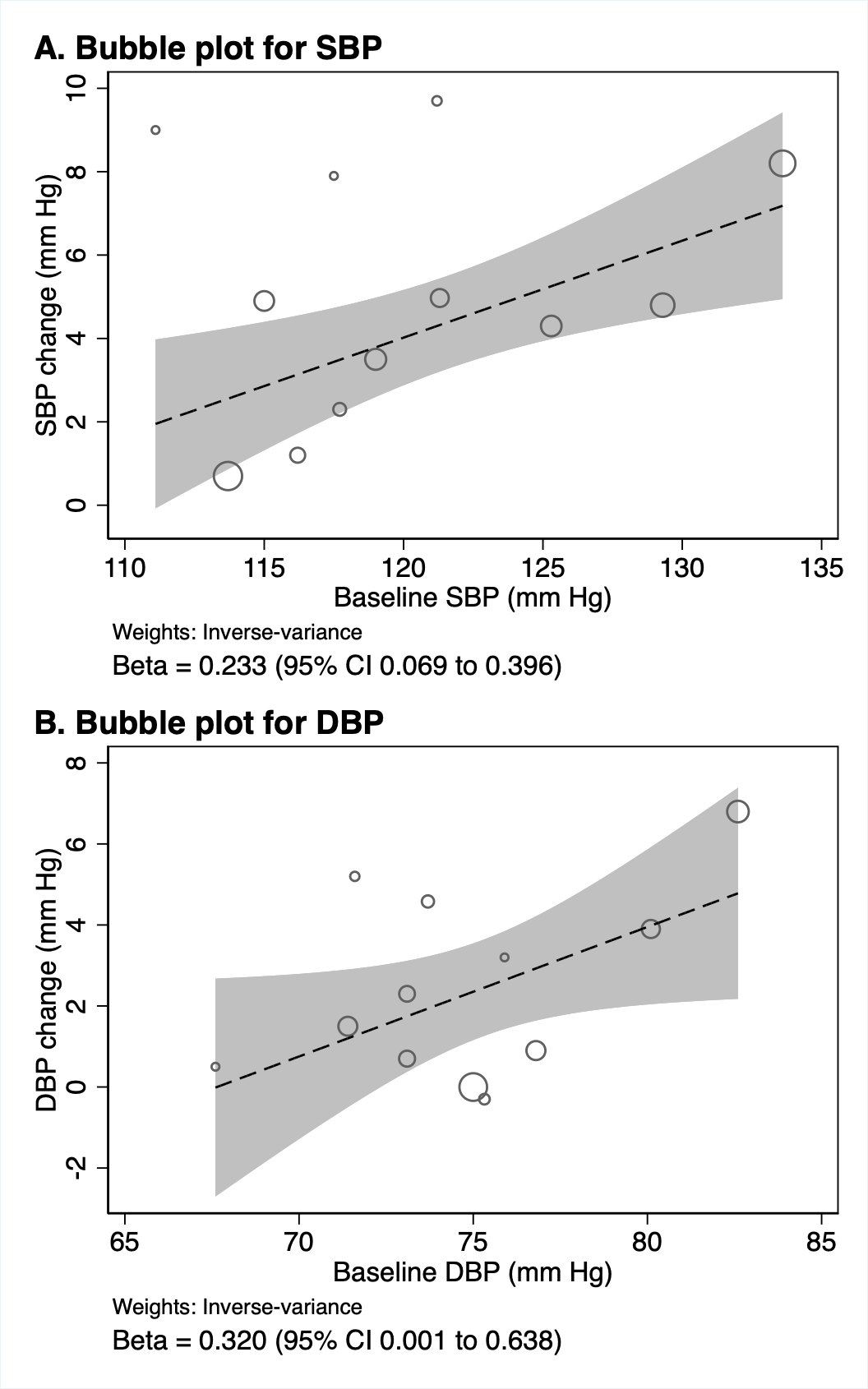
**

**Supplemental Figure S6.** Bubble plots with meta-regression analysis between duration of follow-up on (systolic blood pressure) and DBP (diastolic blood pressure) changes related to usual alcohol intake at baseline ^11, 20, 21, 23-26^. Hole circles (“bubbles”) are proportional to study weights. Dash lines indicates the predicted regression line with grey areas of confidence-interval bands.

**

**Supplemental Figure S7.** Funnel-plots for publication bias the highest versus the lowest alcohol intake and systolic blood pressure (SBP) and diastolic blood pressure (DBP) ^11, 20, 21, 23-26^. MD: mean difference; CI: confidence interval; SE: standard error.
